# Glucosamine facilitates cardiac ischemic recovery via recruiting Ly6C^low^ monocytes in a STAT1 and O‐GlcNAcylation‐dependent fashion

**DOI:** 10.1002/ctm2.762

**Published:** 2022-03-28

**Authors:** Wenjing Zhou, Xuan Jiang, Qingsong Tang, Liang Ding, Weizhang Xiao, Jingjing Li, Yong Wu, Hai‐Bin Ruan, Zhenya Shen, Weiqian Chen

**Affiliations:** ^1^ Department of Cardiovascular Surgery of the First Affiliated Hospital & Institute for Cardiovascular Science Suzhou Medical College of Soochow University Soochow University Suzhou China; ^2^ Molecular Cancer Research Center School of Medicine Sun Yat‐Sen University Shenzhen China; ^3^ Department of Integrative Biology and Physiology University of Minnesota Medical School Minneapolis Minnesota USA; ^4^ Center for Immunology University of Minnesota Medical School Minneapolis Minnesota USA

Dear Editor,

*O*‐linked β‐*N*‐acetylglucosamine (*O*‐GlcNAc) modification controls a variety of biological processes. Though sustained hyper‐*O*‐GlcNAcylation aggravates pathogenesis of many chronic diseases, accumulating evidence also indicates that acute augmentation in *O*‐GlcNAcylation seems to benefit disease healing in some cases.^1^ Glucosamine (GlcN, 2‐amino‐2‐deoxy‐d‐glucose) is a freely available and commonly used dietary supplement for human cartilage health,^2^ which also activates hexosamine biosynthesis pathway and induces protein *O*‐GlcNAcylation.^3^ In the present study, we attempted to induce acute hyper‐*O*‐GlcNAcylation by GlcN for ischemic repair and further dissect its underlying mechanisms. Notably, GlcN early therapy (GlcN/E), which initiated 1 day before myocardial infarction (MI), effectively facilitated cardiac ischemic recovery, as evidenced by the enhanced heart function (Figure 1A–C), smaller heart weight (Figure 1D,E) and restricted scar formation (Figure 1F–I). More importantly, short‐term GlcN therapy initiated even 3 days post‐MI (GlcN/L) was also sufficient to induce clear cardiac protection (Figure 1A–I), suggesting that both GlcN/E and GlcN/L therapies effectively ameliorate post‐MI cardiac dysfunction and scar formation.

**FIGURE 1:**
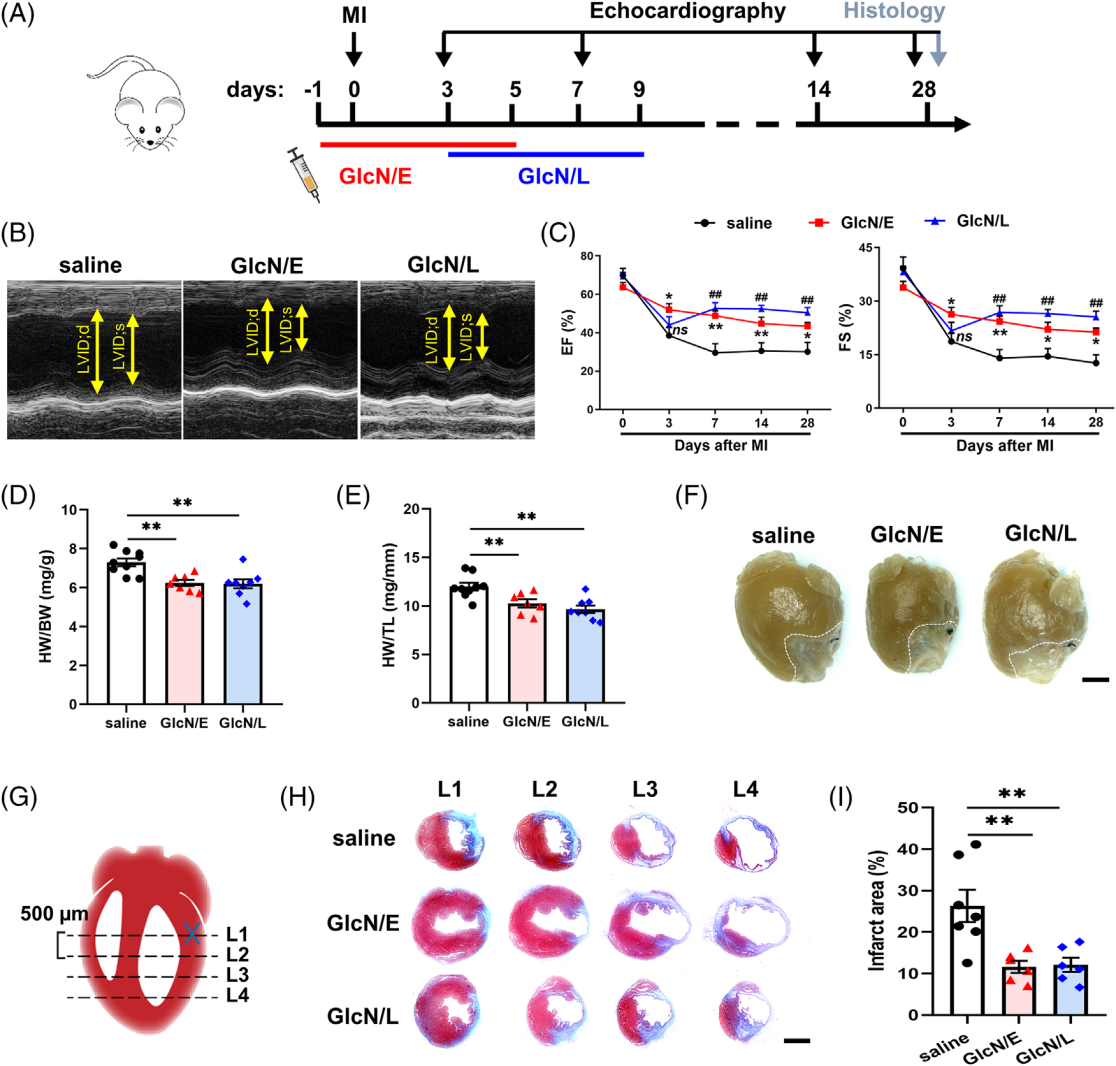
GlcN administration attenuates post‐myocardial infarction (MI) cardiac dysfunction and infarct size in mice. (A) Schedule illustrating early and late GlcN therapies. (B) Representative echocardiography images on Day 28 post‐MI. (C) Time course of ejection fraction (EF) and fractional shortening (FS) after indicated treatments (*n* = 6–9). *, ** indicates saline vs. GlcN/E; ## indicates saline vs. GlcN/L. (D–F) heart weight normalized to body weight (HW/BW) (D) or tibia length (HW/TL) (E) and gross observations (F) on Day 28 post‐MI (*n* = 7–9). Scale bar = 2 mm. (G) Heart schematic pinpointing location of ligation (cross) and sampling (dashed line). Serial sectioning was performed at 500 μm intervals. (H) Representative images for Masson's trichrome staining on Day 28 post‐MI. Scale bar = 2 mm. (I) Quantitation of infarct area (*n* = 6–7). HW, heart weight (mg); BW, body weight (g); TL, tibia length (mm). Data are represented as mean ± SEM. *ns* not significant, *P < 0.05, **P < 0.01, ##*P* < 0.01 by two‐way ANOVA followed with Tukey's multiple comparisons test (C) or by one‐way ANOVA followed with Tukey's multiple comparisons test (D, E and I)

RNA‐seq was performed to obtain an unbiased, genome‐wide view of GlcN‐mediated cardiac protection. Totally 602 genes were twofold dysregulated after GlcN/E therapy, with 295 down‐regulated and 307 up‐regulated (Figure 2A), and genes related to the immune system, infectious diseases and immune diseases were especially enriched among all the dysregulated genes (Figure 2B). As an essential cellular protagonist in innate immunity, Monocyte/Macrophages (Mo/Mps) play both beneficial and detrimental roles during the ischemic wound healing process.^4^ Accordingly, much more Ly6C^low^ Mps, which support tissue regeneration, were captured by flow cytometry after GlcN therapy, whereas Ly6C^high^ Mps, which scavenge debris and exacerbate inflammation, remained comparable between the two groups (Figure 2C–E). Consistently, T cell accumulation also remained similar between groups (Figure S1A,B). Ly6C^low^ and Ly6C^high^ Mo/Mps are generally considered as reparative and inflammatory subpopulations.^5^ To directly visualize in vivo accumulation of these two subpopulations, immunofluorescent staining was performed and more reparative (CD206^+^), but comparable total (CD68^+^) and inflammatory (iNOS^+^) Mps were observed in GlcN‐treated ischemic myocardium (Figure 2F), indicating that GlcN calms hyper‐inflammation via accumulating predominantly reparative Mps.

**FIGURE 2:**
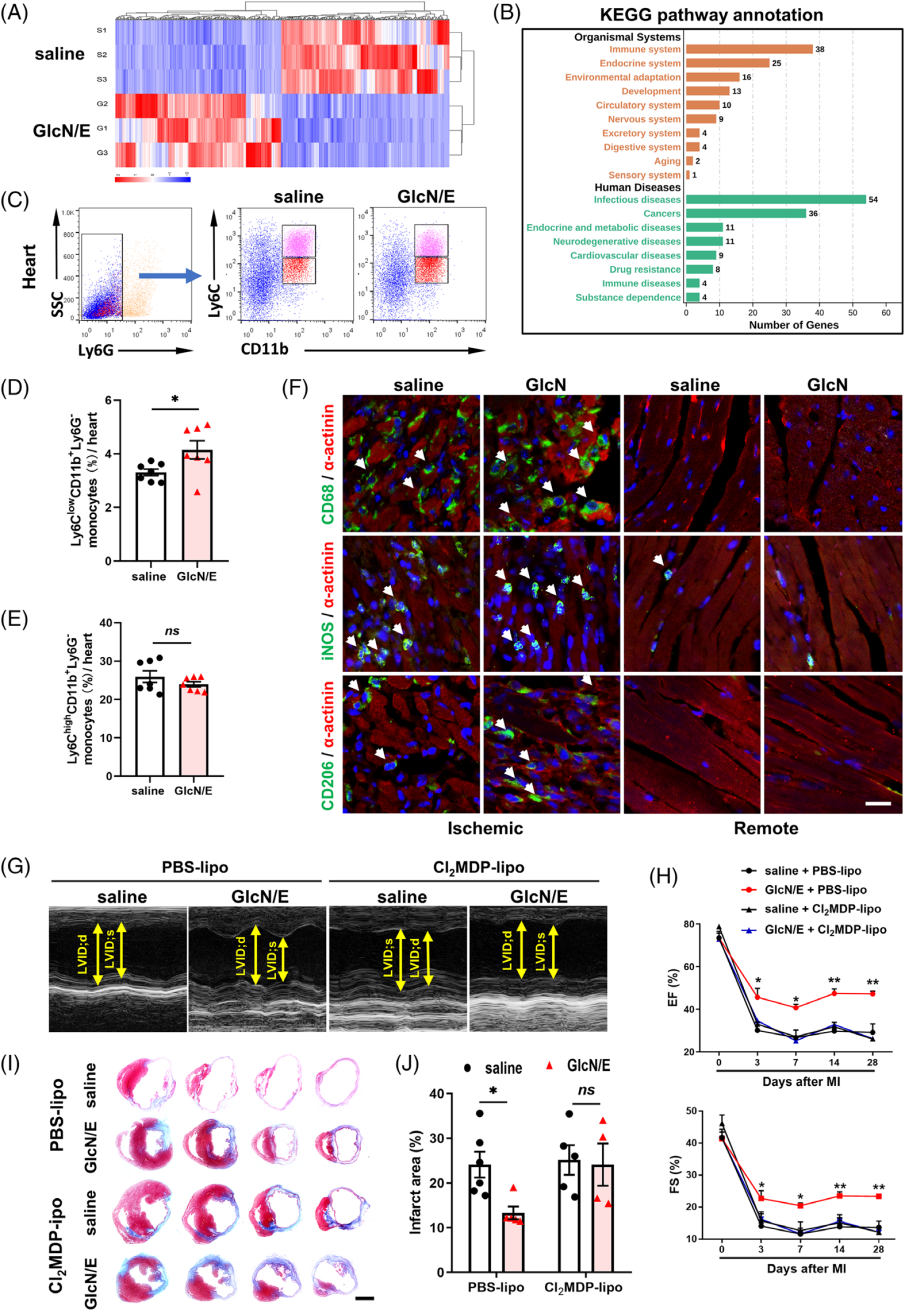
GlcN preserves post‐ myocardial infarction (MI) cardiac function by primarily targeting Mo/Mps. (A) Hierarchical clustering of 602 differentially expressed genes between saline and GlcN/E hearts 3 days after MI. Red and blue colours indicate up‐regulated or down‐regulated genes. (B) KEGG analysis of 602 dysregulated genes. (C–E) Representative plots and quantification of Ly6C^low^ reparative (Ly6C^low^CD11b^+^Ly6G^–^) and Ly6C^high^ inflammatory (Ly6C^high^CD11b^+^Ly6G^–^) Mo/Mps in ischemic myocardium on Day 3 post‐MI (*n* = 7). (F) Representative images of CD68^+^, iNOS^+^ and CD206^+^ cells on Day 3 post‐MI. Scale bar = 20 μm. (G) Representative echocardiography images of infarcted hearts on Day 28 post‐MI. (H) Post‐MI cardiac function with indicated treatments (*n* = 4–8). *, ** indicates saline + PBS‐lipo *vs* GlcN/E + PBS‐lipo. (I&J) Masson's trichrome‐stained hearts on Day 28 post‐MI and quantitation of infarct area (*n* = 4–6). Scale bar = 2 mm. PBS‐lipo, PBS liposomes; Cl_2_MDP‐lipo, clodronate liposomes. Data are represented as mean ± SEM. *ns* not significant, *P < 0.05, **P < 0.01 by two‐tailed unpaired Student's *t* test (D and E) or by two‐way ANOVA followed with Tukey's multiple comparisons test (H and J)

In the setting of Mo/Mps removal (Figure S2A,B), however, neither GlcN/E nor GlcN/L therapy was able to maintain their resistance to post‐MI cardiac dysfunction and myocardium loss (Figure 2G–J, Figure S3A–C), confirming requirement of Mo/Mps in the GlcN therapy. On the other side, angiogenetic behaviour of HUVECs (Figure S4A–G), ROS production by hypoxic cardiomyocytes and proliferation of cardiac fibroblasts (Figure S5A–C) all remained unaltered after the GlcN treatment, excluding the possibility that other cell types may also benefit infarct repair by GlcN.

Mo/Mps can be typically classified into two distinct subpopulations: inflammatory and reparative.^4^ For LPS/IFN‐γ‐stimulated inflammatory activation, protein or mRNA levels of iNOS, CD80, CD86, *Il1b*, *Il6*, *Nos2*, *Tnf*, *Cd80* and *Il12p40* all remained unchanged despite GlcN exposure (Figure S6A–E). For IL‐4‐induced reparative activation, GlcN also failed to alter protein or mRNA levels of most signature genes, including CD206, *Relma*, *Mgl2*, *Ym1*, *Fabp4* and arginase 1 (Figure S6F–H). In sum, our data indicated that neither inflammatory nor reparative activation is altered in response to GlcN, suggesting that GlcN acts independently of dynamic activation of Mo/Mps.

Aside from dynamic activation, recruitment of diverse Mos also judges tissue repair and C‐X3‐C ligand 1 (CX3CL1)‐dependent infiltration of Ly6C^low^ reparative Mos is known to calm ischemic hyper‐inflammation.^6^, ^7^ Consequently, both IL‐4‐elicited and IL‐13‐activated immortalized bone marrow‐derived macrophages (iBMDMs) exhibited augmented chemotaxis towards CX3CL1 (Figure 3A). Consistent with the above in vitro findings, although *Cx3cl1* expression in ischemic myocardium remained unchanged (Figure 3B), much more Ly6C^low^ Mos in peripheral blood were observed after GlcN therapy (Figure 3C,D). CX3CL1‐initiated chemotaxis depends largely on C‐X3‐C receptor 1 (CX3CR1).^6^ Accordingly, GlcN‐induced considerable elevation in CX3CR1 expression (Figure 3E, Figure S9A) and chemical blockade by CX3CR1 neutralizing antibody (CX3CR1‐Ab) or genetic silencing by siCX3CR1 both abolished pro‐chemotaxis effect of GlcN (Figure 3F,G, Figure S7A,B). Not surprisingly, administering CX3CR1‐Ab *in vivo* completely abolished the cardioprotective effect of GlcN (Figure 3H–L). Therefore, GlcN supports infiltration of Ly6C^low^ reparative Mos through upregulation of CX3CR1.

**FIGURE 3:**
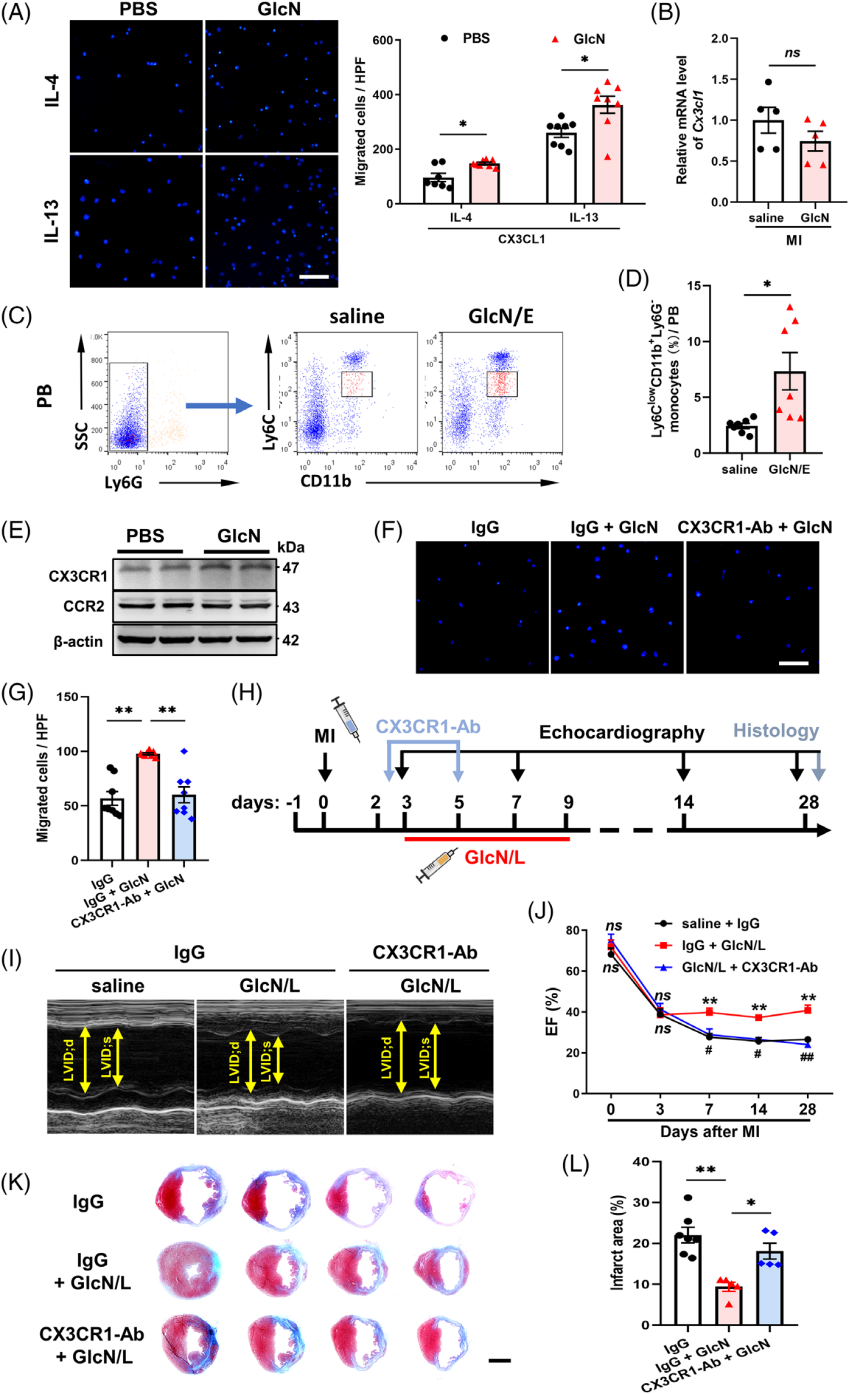
GlcN enhances ischemic infiltration of Ly6C^low^ Mos via positively regulating myeloid *Cx3cr1* gene transcription. (A) iBMDM cells treated with IL‐4 ± GlcN or IL‐13 ± GlcN were subjected to in vitro transwell assay (*n* = 7–8). Scale bar = 100 μm. (B) *Cx3cl1* mRNA expression of ischemic myocardium on Day 3 post‐MI (*n* = 3–4). (C,D) Representative plots and quantification of Ly6C^low^ reparative Mos (Ly6C^low^CD11b^+^Ly6G^–^) in peripheral blood (PB) on Day 3 post‐myocardial infarction (MI) (*n* = 7). (E) Western blot analysis of CX3CR1 and CCR2 on iBMDMs treated with IL‐4 ± GlcN. (F,G) IL‐4‐elicited iBMDMs were incubated with CX3CR1 neutralizing antibody (CX3CR1‐Ab) and subjected to in vitro transwell assay (*n* = 8). Scale bar = 100 μm. (H) Strategy of CX3CR1‐Ab infusion on post‐MI ischemic healing. (I) Representative echocardiography images on Day 28 post‐MI. (J) Time course of post‐MI EF with indicated treatments (*n* = 6–9). ** indicates saline + IgG vs. GlcN/L+ IgG, #, ## indicates GlcN/L+ IgG vs. GlcN/L + CX3CR1‐Ab. (K,L) Masson's trichrome‐stained hearts on Day 28 post‐MI and quantitation of infarct area (*n* = 5–7). Scale bar = 2 mm. PB, peripheral blood. Data are represented as mean ± SEM. *ns* not significant, *P < 0.05, **P < 0.01, #*P* < 0.05, ##*P* < 0.01 by two‐tailed unpaired Student's *t* test (A, B, D), or by one‐way ANOVA followed with Bonferroni's multiple comparisons test (G, L), or by two‐way ANOVA followed with Tukey's multiple comparisons test (J)

We next tried to figure out whether and at which level protein *O*‐GlcNAcylation controls the pro‐chemotaxis effect of GlcN. First, GlcN dramatically elevated myeloid *O*‐GlcNAcylation (Figure 4A,B) and pharmacological inhibition of *O*‐GlcNAc transferase by OSMI‐1 fully negated this pro‐chemotaxis effect, even resulting in worse mobility (Figure S8, Figure 4C). Most STAT proteins can be *O*‐GlcNAc‐modified based on existing reports.^8^ We have previously demonstrated that STAT1, which positively modulates myeloid *Cx3cr1* transcription,^9^ can be *O*‐GlcNAc‐modified.^8^ Consistently, myeloid *Cx3cr1* transcription was also elevated following GlcN supplementation (Figure 4D). We hence questioned whether GlcN augments *Cx3cr1* transcription via STAT1 *O*‐GlcNAcylation. Accordingly, *O*‐GlcNAcylation, protein quantity and nuclear translocation of STAT1 were all greatly elevated in GlcN‐treated iBMDMs (Figure 4E–G, Figure S9B), indicating that *O*‐GlcNAcylation may control quantity and nuclear translocation of STAT1. Notably, co‐treatment of GlcN‐elicited iBMDMs with OSMI‐1 successfully diminished STAT1 protein (Figure 4H, Figure S9C) and *Cx3cr1* mRNA level (Figure 4I). Finally, GlcN's ability to accelerate chemotaxis was also blocked after STAT1 knockdown (Figure 4J,K, Figure S9D), confirming that GlcN is functioning at the STAT1 level, though other functional molecules may also exist. Altogether, these results suggest that GlcN promotes *Cx3cr1* gene transcription via STAT1 *O*‐GlcNAcylation.

**FIGURE 4:**
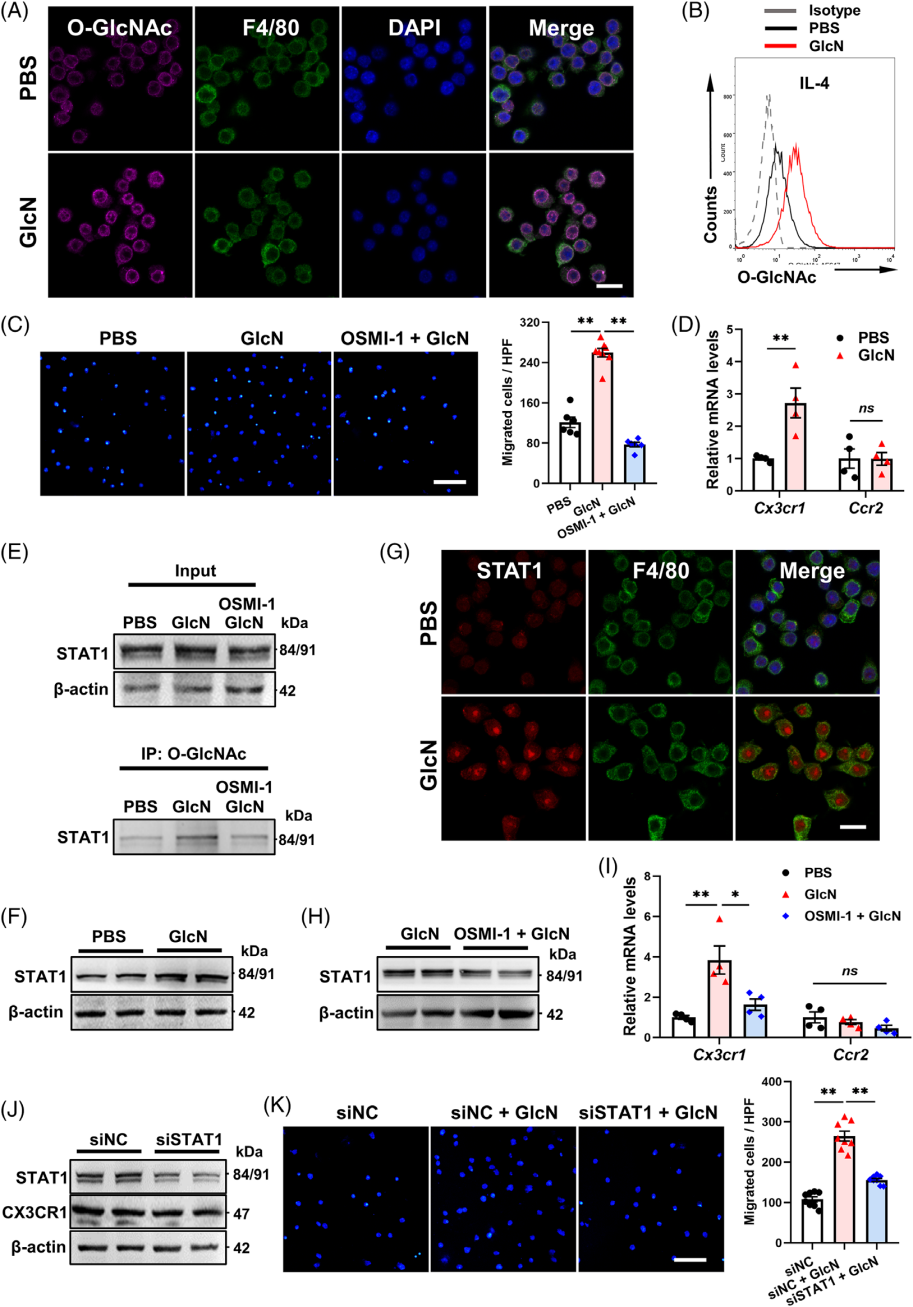
GlcN stabilizes STAT1 by mediating *O*‐GlcNAcylation of STAT1. (A,B) iBMDMs were incubated with IL‐4 ± GlcN for 24 h. (A) Representative immunostaining images of *O*‐GlcNAc, F4/80 and DAPI. Scale bar = 10 μm. (B) Flow cytometry analysis for *O*‐GlcNAc level. (C) iBMDMs stimulated with indicated treatments were subjected to in vitro transwell assay (*n* = 6–8). Scale bar = 100 μm. (D–G) iBMDMs were incubated with IL‐4 ± GlcN for 24 h. (D) mRNA expression profiles of *Cx3cr1* and *Ccr2* (*n* = 4). (E) *O*‐GlcNAcylation level of STAT1 with indicated treatments. (F) Western blot analysis of STAT1. (G) Representative immunostaining images of STAT1, F4/80 and DAPI. Scale bar = 10 μm. (H,I) iBMDMs were incubated with GlcN ± OSMI‐1 for 24 h. (H) Western blot analysis of STAT1. (I) mRNA expression profiles of *Cx3cr1* and *Ccr2* (*n* = 4). (J,K) Effect of STAT1 silencing on migration of IL‐4‐elicited Mo/Mps toward CX3CL1 (*n* = 8). Scale bar = 100 μm. HPF, high‐power fields. Data are represented as mean ± SEM. *ns* not significant, *P < 0.05, **P < 0.01 by one‐way ANOVA followed with Bonferroni's multiple comparisons test (C, I, K) or by two‐tailed unpaired Student's *t* test (D)

We have previously demonstrated that manipulating aspartate–arginosuccinate shunt of inflammatory Mo/Mps alters their inflammatory response and hence accelerates cardiac healing.^10^ Here, we show that short‐term hyper‐*O*‐GlcNAcylation by pre‐MI infusion of GlcN, a GLP‐approved diet supplement, effectively facilitates in vitro chemotaxis of reparative Mo/Mps and in vivo infiltration of Ly6C^low^ Mos, leading to accelerated cardiac recovery. Intriguingly, GlcN therapy initiated even 3‐days post‐MI is also sufficient to produce clear cardiac protection. Mechanistically, GlcN positively regulates myeloid *Cx3cr1* transcription by improving STAT1 *O*‐GlcNAcylation, which subsequently promotes nuclear translocation and transcriptional activity of STAT1 (Figure S10).

## CONFLICT OF INTEREST

The authors declare that there is no conflict of interest that could be perceived as prejudicing the impartiality of the research reported.

## Supporting information

Supporting information

## Data Availability

RNA‐Seq data: Gene Expression Omnibus GSE193613.
