# Supplementary material for: Glucosamine facilitates cardiac ischemic recovery via recruiting Ly6Clow monocytes in a STAT1 and O‐GlcNAcylation‐dependent fashion
Source: Clin Transl Med. 2022 Mar 28;12(3):e762. doi: 10.1002/ctm2.762 (PMC8958344; doi:10.1002/ctm2.762)
Supplement: Supplementary file 1 — Supporting information [file CTM2-12-e762-s001.pdf]

Supplemental Materials

Materials and methods

Ethics statement

All animal procedures were approved by the Animal Use Subcommittee of Soochow University, China and conformed to Guide for the Care and Use of Laboratory Animals (National Institutes of Health publication No. 85-23, revised 1996).

Animals and treatments

Daily injection of GlcN (Sigma, 0.3 g/kgBW) was intraperitoneally administered for ~~6~~ 7 consecutive days. For GlcN/E therapy, GlcN injection begins 1 d before MI; whereas for GlcN/L therapy, GlcN injection begins 3 d after MI as described previously.[1] Depletion of Mo/Mps was achieved by Cl_2MDP-lipo administration (ClodronateLiposomes.org, 250 μg/mice/injection) with a total of 2 intraperitoneal injections every 3 days as described previously.[2,3] The same volume of PBS liposomes (PBS-lipo) was injected in control group. For CX3CR1 neutralizing antibody blockade, 20 μg of rabbit IgG or rabbit anti-CX3CR1 antibodies (Torrey Pines Biolabs) were injected intravenously by tail vein 3 d after MI and every other day thereafter for a total of 4 days as described previously.[4]

Cell culture, treatments, and gene silencing

C57BL/6 mice-derived immortalized bone marrow-derived macrophages (iBMDM) cells were kindly provided by Feng Shao (National Institute of Biological Sciences, China) and maintained in RPMI 1640 medium (Procell) supplemented with 10% fetal bovine serum (FBS, ExCell Bio). iBMDMs were activated to inflammatory status with LPS (Sigma, 20 ng/mL) plus IFN-γ (PeproTech, 20 ng/mL) or to reparative status with IL-4 (PeproTech, 40 ng/mL) or IL-13 (Cyagen Biosciences, 20 ng/mL) for 24 h. Human umbilical vein endothelial cells (HUVECs) were cultured in Endothelial Growth Medium-2 (EGM-2; Lonza) containing complete supplements. Primary neonatal mouse ventricular myocytes (NMVMs) and cardiac fibroblasts were isolated and cultured as we previously described.[8] For hypoxic treatment, cells were cultured in a tri-gas incubator composed of 1% O_2, 94% N_2, and 5% CO_2 for 48 h.

GlcN (Sigma, 3 mM), OSMI-1 (Sigma, 50 μM), and CX3CR1 neutralizing antibody (Torrey Pines Biolabs, 20 μg/mL) were used when indicated. siRNAs were transfected by Lipofectamine RNAiMAX (Invitrogen) according to the manufacturer's protocol.[5] Efficiency of siRNA-mediated gene silencing was evaluated by western blot analysis. Sequences of specific

siRNAs are described below: siCX3CR1 mix: 5'-CCUGUUAUUUGGGCGACAUTT-3', 5'-CCCAGUUCAUGUUCACAAATT-3', and 5'-GUUCAGAAGAUACCUGGGATT-3'; siSTAT1: 5'-AAGGAAAAGCAAGCGTAATCT-3' (GenePharma).

Transwell assay

Migration analysis was carried out using the Boyden transwell chambers (Corning, 8.0-μm pore size) as we described previously.[6] Briefly, stimulated cells were seeded at 2×10^5 cells/100 μL in the upper chamber of a 24-well plate, and 500 μL medium containing CX3CL1 (PeproTech, 20 ng/mL) and FBS (2%) was added into the lower chamber. After 4 h incubation, remaining cells on upper side of the insert were wiped off and cells that migrated to the reverse side of the insert were stained with DAPI and counted.

Tube formation assay

Angiogenic ability of HUVECs was assessed by tube formation assay as we described previously.[7] Briefly, 100 μL thawed Matrigel (BD) was coated in 96-well plates at 37°C to form a reconstituted basement membrane. Next 2×10^4 stimulated cells in 100 μL EGM-2 were seeded on the Matrigel and incubated at 37°C for 6 h. Tube structures were then inspected under an inverted light microscope and analyzed by Image J software. Tube formation was determined by measuring closed network/high power fields (HPF), tube length/HPF, and vascular branches/node.

Wound-healing assay

Wound-healing assay was performed as we described previously.[6] Briefly, wounds were made with a sterile pipette tip on HUVECs when >90% confluency and dislodged cells were washed away. Wounds were photographed on 0, 12, and 24 h post-wounding. Healing percentage was defined as difference between the original and remaining wound areas, and is expressed as a percentage of the original area.

Cell proliferation and ROS accumulation assay

Cell proliferation was determined with an EdU *In Vitro* Imaging kit (Beyotime) according to the manufacturer's instructions.[7] Briefly, cells were incubated with 10 μM ethynyldeoxyuridine (EdU) for 2h. Nuclear EdU was further marked by binding of Alexa Fluor 594-labeled azide to its alkyne group. Then, the cells were stained with DAPI (Yeasen) and EdU incorporation was counted randomly per high power fields in each well. Intracellular ROS production was fluorometrically monitored using 2',7'-dichlorofluorescein diacetate (DCFH-DA) (Yeasen) as we

previously described,[8] and images were monitored under inverted fluorescence microscope (Olympus).

Myocardial infarction

Permanent MI was performed on male C57BL/6 mice by ligation of left anterior descending (LAD) artery as we previously described.[7,8] Briefly, animals were put under general anesthesia and ventilated by a rodent respirator. After a left thoracotomy between the third and fourth intercostal space, the left ventricle was exposed satisfactorily and LAD was ligated. Successful induction of MI was verified by a color change in the infarct region after ligation.

Echocardiography

Echocardiography was performed using a Vevo 2100 high-resolution imaging system equipped with a 30-MHz transducer (VisualSonics) as we previously described.[7,8] End diastole was measured at the time of the apparent maximal LV diastolic dimension, and end systole was measured at the time of the most anterior systolic excursion of the posterior wall.

Isolation of cardiac immune cells for flow cytometry

Ischemic myocardium was rapidly removed, minced, and digested for 80 min with prewarmed buffer containing 2% collagenase type 2 (Worthington), 0.25% elastase (Worthington), and 0.05% DNase I (Sigma) as previously described.[9] Digested cells were filtered through a 70-µm cell strainer after the enzymatic reaction.

Flow cytometry

For surface markers, cells were stained in PBS containing 2% FBS with relevant antibodies for 30 min. For analysis of intracellular markers, cells were first fixed with Fixation/Permeabilization buffer (ThermoFisher) and then stained in Permeabilization Buffer (ThermoFisher) with relevant antibodies for 30 min. For tissue cells, red blood cells were lysed before antibody staining. Corresponding fluorescent conjugated isotype controls (MultiSciences) were also used in each staining panel. Flow cytometry data were acquired on Millipore Guava easyCyte and analyzed using FlowJo software. All antibodies have been listed in Supplementary Table 1. Ly6C^{low} reparative and Ly6C^{high} inflammatory Mo/Mps were identified as Ly6C^{low}CD11b^{+}Ly6G^{-} and Ly6C^{high}CD11b^{+}Ly6G^{-}, respectively.

Histology and Masson's trichrome staining

Mouse hearts were arrested with diastole buffer (10% KCl) and fixed in 4% paraformaldehyde solution sequentially. Infarct area was analyzed with Masson's trichrome-stained images

(Solarbio) and quantified using Image J software as we described previously.[10] Briefly, ischemic hearts were transversely sectioned from point of ligation to apex of the heart. Serial sections were collected every 500 μm intervals. The percentage of infarct size was calculated as scar area/total LV area × 100%.

Immunostaining

For fluorescent immunohistochemistry, cells grown on coverslips or frozen tissue sections were routinely fixed, permeabilized, and blocked as we described previously.[8] Slides were further incubated with primary antibodies (1:100 to 1:200 dilution) overnight and fluorescent conjugated secondary antibodies (Yeasen). Nuclei were counterstained with DAPI (Yeasen). Images were captured using a confocal microscope (Zeiss, LSM880) and processed using ZEN software. All antibodies have been listed in Supplementary Table 1.

RNA extraction, Reverse transcription (RT) -PCR, and quantitative (q) RT-PCR

Total RNA was extracted based on phenol-chloroform method, quantified using NanoDrop 2000 spectrophotometer, and reversed transcribed with HiScript III RT SuperMix Kit (Vazyme). qRT-PCR was carried out using SYBR Premix Ex Taq reaction mix (Takara) on StepOne Plus real-time PCR system (Applied Biosystems) as we previously reported.[11] Expression of target genes was determined by comparative ΔΔCt method and *18S* was used as an internal control gene. Primer sequences are provided in Supplementary Table 2.

Immunoprecipitation and western blot analysis

Briefly, cells after indicated treatments were lysed using RIPA buffer containing proteinase inhibitors, protein phosphatase inhibitors and thiamet-G (Sigma) before quantitated with a BCA assay kit (Novoprotein). For immunoprecipitation, whole-cell lysates were incubated with anti-*O*-GlcNAc (Abcam, ab2739, 1:100) or immunoglobulin G and precipitated with protein A/G agarose beads (Senta Cruz) overnight at 4°C. Equal amounts of protein lysates or immunoprecipitated samples were processed for western blot analysis following standard protocol as we described previously.[12] The following primary antibodies were used: CX3CR1 (1:2000), CCR2 (1:1000), STAT1 (1:1000), and β-actin. Immunoreactivity was detected by routine enzymatic chemiluminescence (Meilun Biotechnology). All antibodies have been listed in Supplementary Table 1.

RNA-seq and genome-wide transcriptome analysis

Ischemic zones from GlcN/E or saline hearts on Day 3 post-MI (three biological replicates per group) were collected in TRIzol reagent for RNA-seq (Novogene). Briefly, mRNA was purified from total RNA using poly-T oligo-attached magnetic beads and subjected to library preparation using NEBNext UltraTM RNA Library Prep Kit for Illumina (NEB). The library preparations were sequenced on an Illumina NovaSeq 6000 sequencer. For data analysis, raw data (raw reads) in fastq format were first processed through FastQC. Both the mouse reference genome (GRCm39) and transcript annotation were downloaded from Ensemble and RNA-seq reads were mapped to the reference genome using Hisat2. After alignment, StringTie was used to assemble aligned reads into transcripts and estimate their abundance. Differential gene expression analysis was performed using the DESeq2 R package and the differentially expressed genes with an absolute fold change > 2 and False Discovery Rate < 0.05 were identified as statistically significant. KEGG pathway analyses were implemented using the clusterProfiler R package to identify significantly enriched terms and the heat map plot and sample clustering were generated using heatmap R package.

Statistics

All data were presented as mean ± SEM as indicated in figure legends. Statistical significance between two groups was determined by unpaired two-tailed Student's *t* test (Microsoft Excel or GraphPad Prism 8). Datasets involving multiple comparisons were assessed by one-way ANOVA with Tukey's correction using GraphPad Prism 8. Multiple comparisons with two independent variables were assessed by two-way ANOVA followed by Tukey's correction using GraphPad Prism 8. *P < 0.05; **P < 0.01; #P < 0.05; ##P < 0.01.

Supplemental Figures

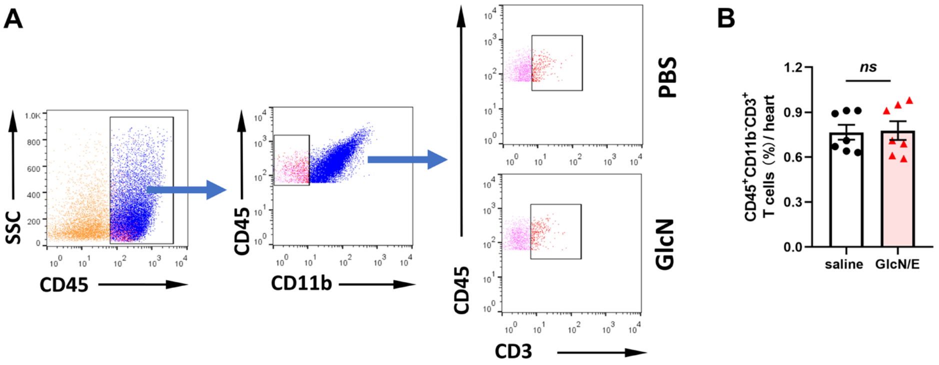

Figure S1 T cell accumulation in ischemic myocardium. Representative plots (A) and quantification (B) of T cells ($CD45^{+}CD11b^{-}CD3^{+}$) in ischemic myocardium on Day 3 post-MI (n = 7). Data are represented as mean ± SEM. *ns* not significant by two-tailed unpaired Student's *t* test.

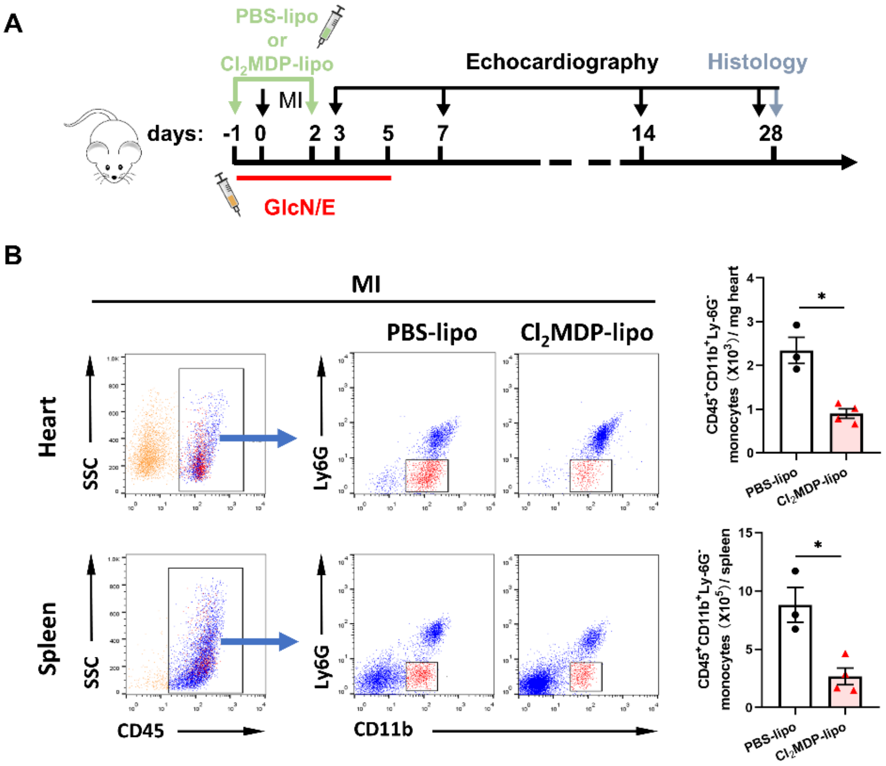

Figure S2 Depletion of Mo/Mps. (A) Schematic diagram of clodronate liposomes (Cl_2MDP-lipo)-induced Mo/Mps depletion model. (B) Flow cytometry analysis was used to determine number of Mo/Mps ($CD45^+CD11b^+Ly6G^-$) in heart and spleen following Cl_2MDP-lipo or PBS-lipo treatment (*n* = 3-4). PBS-lipo, PBS liposomes; Cl_2MDP-lipo, clodronate liposomes. Data are represented as mean ± SEM. **P* < 0.05 by two-tailed unpaired Student's *t* test.

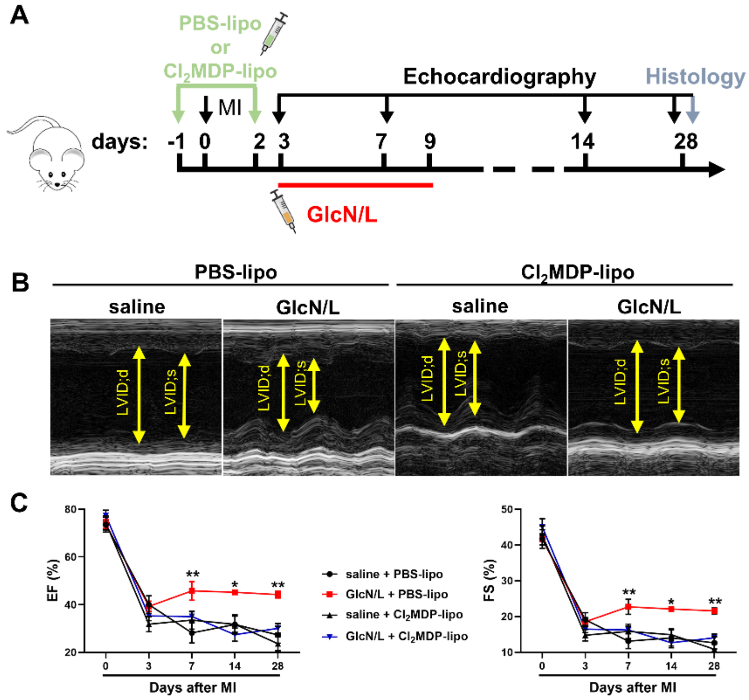

Figure S3 Elimination of Mo/Mps restrains post-MI cardiac healing mediated by GlcN/L therapy. (A) Schematic diagram of Cl_2MDP-lipo-induced Mo/Mps depletion model. (B) Representative echocardiography images of infarcted hearts on Day 28 post-MI following Cl_2MDP-lipo or PBS-lipo treatment. (C) Post-MI cardiac function on Day 0, 3, 7, 14, and 28 with indicated treatments (*n* = 4-8). *, ** indicates saline + PBS-lipo *vs* GlcN/L + PBS-lipo. PBS-lipo, PBS liposomes; Cl_2MDP-lipo, clodronate liposomes. Data are represented as mean ± SEM. *P < 0.05, **P < 0.01 by two-way ANOVA followed with Tukey's multiple comparisons test.

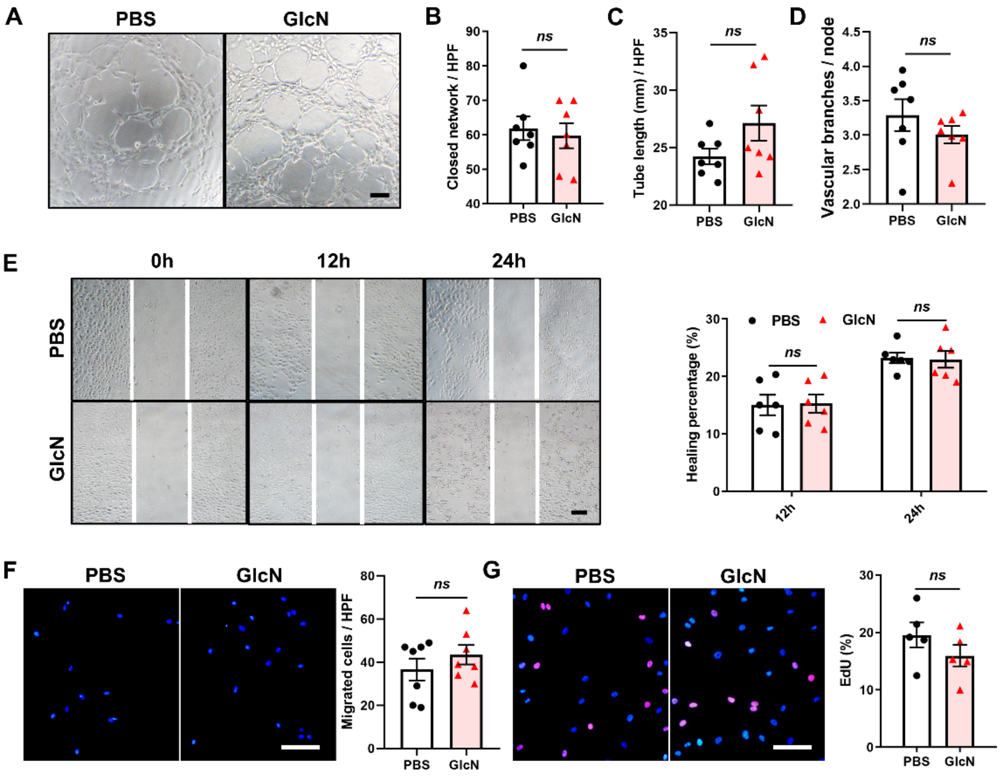

Figure S4 GlcN has no effect on angiogenetic behavior of endothelial cells. Cultured HUVECs were incubated with 3mM GlcN for 24 h. (A-D) Representative images of capillary network on Matrigel and quantification of closed network, tube length and vascular branches (n = 7). Scale bar = 100 µm. (E) Representative images of wound healing assay and healing percentage analysis (n = 6). Scale bar = 200 µm. (F) Transwell assay and quantification of migrated cells per HPF (n = 7). Scale bar = 100 µm. (G) Cell proliferation detected using EdU incorporation assay and percentage of EdU-positive cells (n = 5). Scale bar = 100 µm. HPF, high-power fields. Data are represented as mean ± SEM. *ns* not significant by two-tailed unpaired Student’s t test.

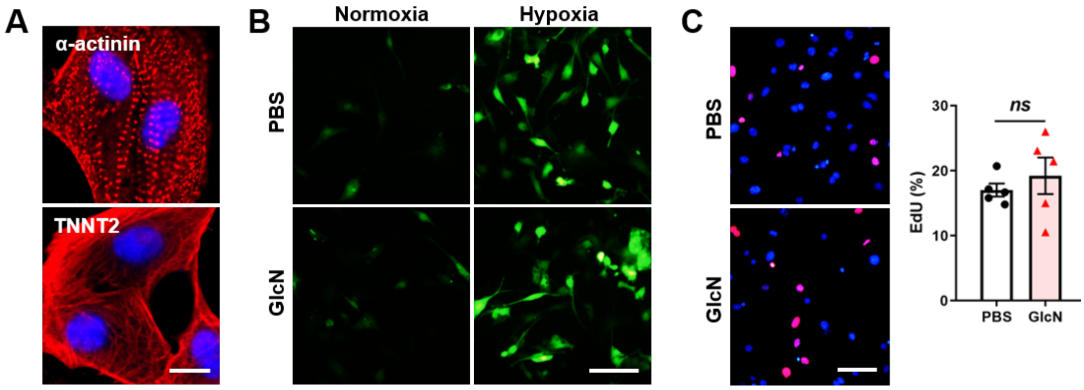

Figure S5 GlcN fails to alter ROS accumulation in cardiomyocytes and proliferation of cardiac fibroblasts. (A) Neonatal mouse ventricular myocytes (NMVMs) were isolated and characterized with cardiac structural makers α-actinin and troponin T (TNNT2). Scale bar = 10 µm. (B) NMVMs were challenged with 1% O_2 for 72h, and intracellular ROS accumulation was monitored with DCF fluorescence. Scale bar = 100 µm. (C) EdU cell proliferation of cardiac fibroblasts and percentage of EdU-positive cells (*n* = 5). Scale bar = 100 µm. Data are represented as mean ± SEM. ns not significant by two-tailed unpaired Student's *t* test.

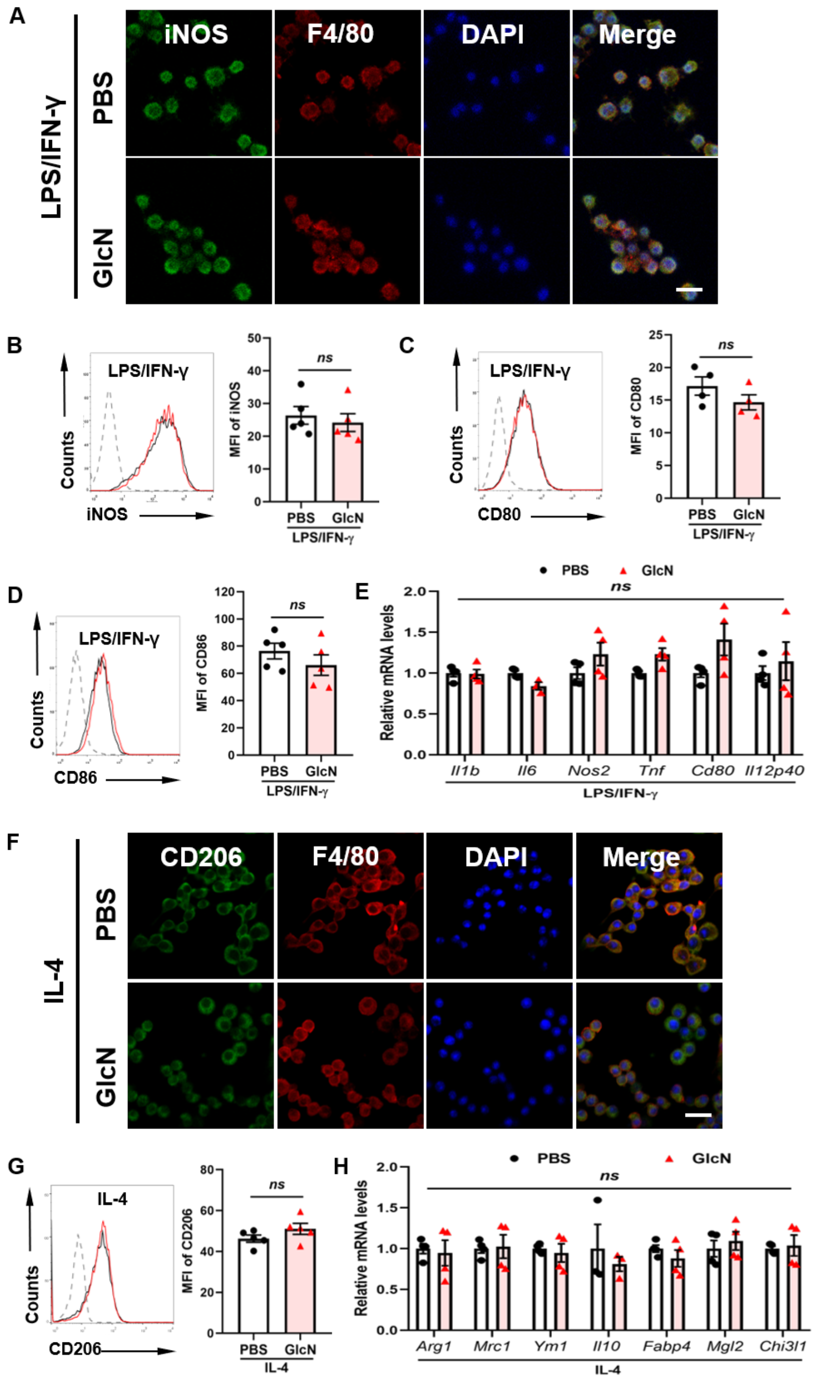

Figure S6 Neither inflammatory nor reparative activation of Mo/Mps is altered by exposure to GlcN. (A-E) iBMDMs were incubated with LPS/IFN-γ ± GlcN for 24 h. (A) Representative immunostaining images of iNOS (green), F4/80 (red) and DAPI. Scale bar = 10 μm. (B-D) Flow cytometry analysis for iNOS, CD80 and CD86 expression (*n* = 4-5). (E) mRNA expression profiles of *Il1b*, *Il6*, *Nos2*, *Tnf*, *Cd80*, and *Il12p40* determined by qRT-PCR (*n* = 3-4). (F-H) iBMDMs were stimulated with IL-4 ± GlcN for 24 h. (F) Representative immunostaining images of CD206 (green), F4/80 (red) and DAPI (blue). Scale bar = 10 μm. (G) Flow cytometry analysis for CD206 expression (*n* = 5). (H) mRNA expression profiles of *Arg1*, *Mrc1*, *Ym1*, *Il10*, *Fabp4*, *Mgl2*, and *Chi3l1* determined by qRT-PCR (*n* = 3-4). MFI, mean fluorescence intensity; *Arg1,* arginase 1. Data are represented as mean ± SEM. *ns* not significant by two-tailed unpaired Student's *t* test.

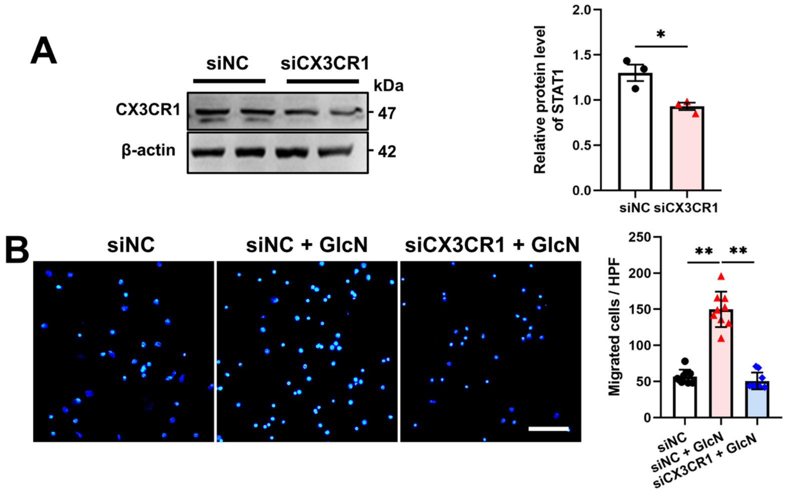

Figure S7 Effect of CX3CR1 silencing on migration of IL-4-elicited Mps in response to CX3CL1. (A&B) IL-4-elicited iBMDMs were transfected with siCX3CR1 or siNC and subjected to western blot analysis or *in vitro* transwell assay (n = 8). Scale bar = 100 µm. Data are represented as mean ± SEM. *$P < 0.05$, **$P < 0.01$ by two-tailed unpaired Student's t test (A) or by one-way ANOVA followed with Bonferroni's multiple comparisons test (B).

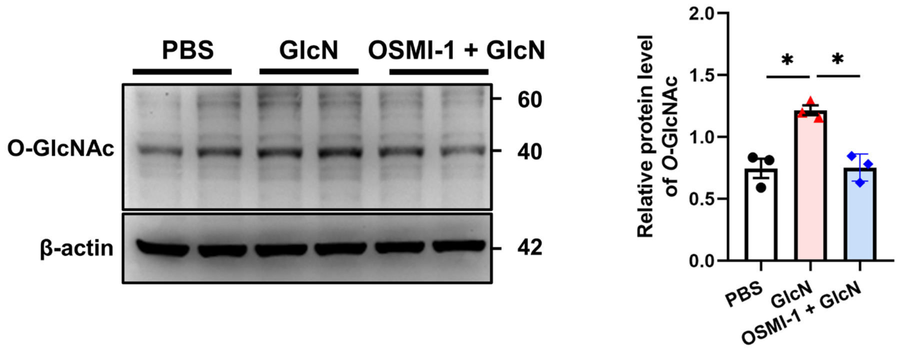

Figure S8 OSMI-1 diminishes protein *O*-GlcNAcylation level. iBMDMs were incubated with indicated treatments for 24 h, and western blot analysis was performed. **P* < 0.05 by one-way ANOVA followed with Bonferroni's multiple comparisons test.

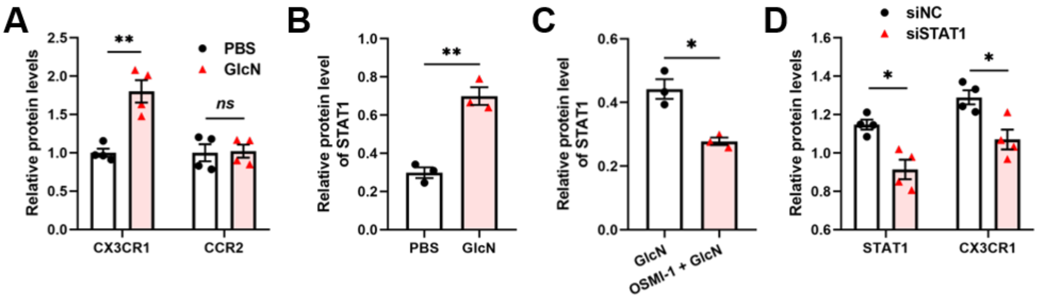

Figure S9 Quantification of western blot analysis. Western blot results were quantitated as follows: (A) Figure 3E (*n* = 4), (B) Figure 4F (*n* = 3), (C) Figure 4H (*n* = 3), and (D) Figure 4J (*n* = 4). Data are represented as mean ± SEM. *P < 0.05, **P < 0.01 by two-tailed unpaired Student's *t* test.

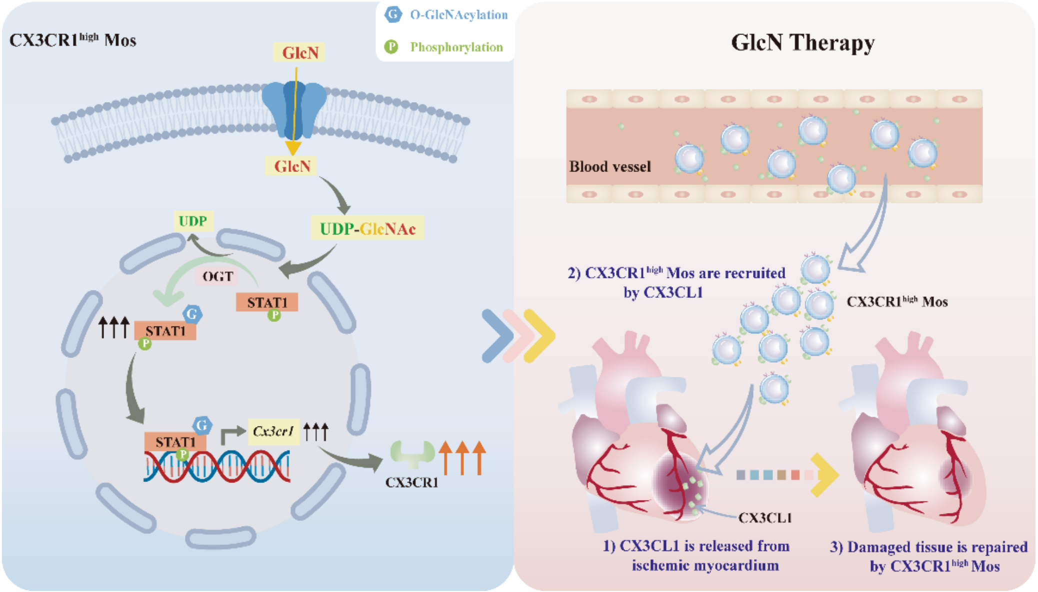

Figure S10 Working model. We have developed a monocyte/macrophages modulation therapy by glucosamine infusion aimed at calming post-MI hyperinflammation in ischemic myocardium. Mechanistically, glucosamine supplementation augments myeloid STAT1 O-GlcNAcylation, which subsequently elevates CX3C chemokine receptor 1 transcription, boosts Ly6C^{low} Mos infiltration, and finally benefits cardiac repair.

Supplementary Table 1 List of antibodies

Target antigen	Source	Catlog
Anti-*O*-GlcNAc	Abcam	ab2739
Anti-OGA	Proteintech	14711-1-AP
Anti-OGT	Proteintech	11576-2-AP
Anti-iNOS	Abcam	ab178945
Anti-CD206	Proteintech	18704-1-AP
Anti-Arg-1	Proteintech	16001-1-AP
Anti-CD68	Abcam	ab53444
Anti-α-actinin	Abcam	ab9465
Anti-Troponin T (TNNT2)	Abcam	Ab8295
Anti-F4/80	Abcam	ab6640
Alexa Fluor 488 anti-iNOS	eBioscience	53-5920-80
Alexa Fluor 488 anti-CD80	BioLegend	104715
FITC anti-CD86	eBioscience	11-0862-81
Alexa Fluor 647 anti-CD206	Abcam	ab195192
PE anti-CD45	eBioscience	12-0451-81
APC anti-CD11b	BioLegend	101211
FITC anti-Ly6G	BioLegend	127605
PE anti-Ly6C	eBioscience	12-5932-80
FITC anti-CD3	BioLegend	100203
Alexa Fluor 647 anti-*O*-GlcNAc	eBioscience	51-9793-41
Anti-CX3CR1	Proteintech	13885-1-AP
Anti-CCR2	Abcam	ab203128
Anti-STAT1	Cell Signaling Technology	14994
Anti-β-actin	Sungene	KM9001T

Supplementary Table 2 List of primer sequences for qRT-PCR

Genes	Forward (5'-3')	Reverse (5'-3')
Il1b	TGTAATGAAAGACGGCACAC	CTCCACTTTGCTCTTGACTTC
Il6	CGTGGACCTTCCAGGATGAG	CATCTCGGAGCCTGTAGTGC
Nos2	GAAGAAAACCCCTTGTGCTG	TCCAGGGATTCTGGAACATT
Tnf	AAACCACCAAGTGGAGGAGC	ACAAGGTACAACCCATCGGC
Cd80	ACCCCCAACATAACTGAGTCT	TTCCAACCAAGAGAAGCGAGG
Il12p40	CACATCTGCTGCTCCACAAGAA	CCAGCCATGAGCACGTGAA
Arg1	CAGAAGAATGGAAGAGTCAG	CAGATATGCAGGGAGTCACC
Mrc1	CTCAACCCAAGGGCTCTTCTAA	AGGTGGCCTCTTGAGGTATGTG
Ym1	AAGAACACTGAGCTAAAAACTCTCCT	GAGACCATGGCACTGAACG
Il10	GCTCTTACTGACTGGCATGAG	CGCAGCTCTAGGAGCATGTG
Fabp4	GGATGGAAAGTCGACCACAA	TGGAAGTCACGCCTTTCATA
Mgl2	AGGCACCCTAAGAGCCATTT	CCCTCTTCTCCAGTGTGCTC
Chi3l1	GATGGCCTCAACCTGGACTG	CGTCAATGATTCCTGCTCCTG
Cx3cl1	ACGAAATGCGAAATCATGTGC	CTGTGTCGTCTCCAGGACAA
Cx3cr1	GAGAGATGGCTCAGTGGTTAG	CACAGGAACAGGGAGCTATTT
Ccr2	TTACACCTGTGG CCCTTATTT	CTGAGTAGCAGATGACCATGAC
18S	GTAACCCGTTGAACCCCATT	CCATCCAATCGGTAGTAGCG

Reference

1. Wang Q, Fang P, He R, Li M, Yu H, Zhou L, et al. O-GlcNAc transferase promotes influenza A virus-induced cytokine storm by targeting interferon regulatory factor-5. *Sci Adv*. 2020;6:eaaz7086.
2. Wang Z, Huang S, Sheng Y, Peng X, Liu H, Jin N, et al. Topiramate modulates post-infarction inflammation primarily by targeting monocytes or macrophages. *Cardiovasc Res*. 2017;113:475-87.
3. Mai CL, Tan Z, Xu YN, Zhang JJ, Huang ZH, Wang D, et al. CXCL12-mediated monocyte transmigration into brain perivascular space leads to neuroinflammation and memory deficit in neuropathic pain. *Theranostics*. 2021;11:1059-78.
4. Nie Y, Huang H, Guo M, Chen J, Wu W, Li W, et al. Breast Phyllodes Tumors Recruit and Repolarize Tumor-Associated Macrophages via Secreting CCL5 to Promote Malignant Progression, Which Can Be Inhibited by CCR5 Inhibition Therapy. *Clin Cancer Res*. 2019;25:3873-86.
5. Chen W, Xia J, Hu P, Zhou F, Chen Y, Wu J, et al. Follistatin-like 1 protects cardiomyoblasts from injury induced by sodium nitroprusside through modulating Akt and Smad1/5/9 signaling. *Biochem Biophys Res Commun*. 2016;469:418-23.
6. Chen W, Wang S, Xia J, Huang Z, Tu X, Shen Z. Protein phosphatase 2A plays an important role in migration of bone marrow stroma cells. *Mol Cell Biochem*. 2016;412:173-80.
7. Shen H, Cui G, Li Y, Ye W, Sun Y, Zhang Z, et al. Follistatin-like 1 protects mesenchymal stem cells from hypoxic damage and enhances their therapeutic efficacy in a mouse myocardial infarction model. *Stem Cell Res Ther*. 2019;10:17.
8. Xiao Y, Zhang Y, Chen Y, Li J, Zhang Z, Sun Y, et al. Inhibition of MicroRNA-9-5p Protects Against Cardiac Remodeling Following Myocardial Infarction in Mice. *Hum Gene Ther*. 2019;30:286-301.
9. Wang J, Liu M, Wu Q, Li Q, Gao L, Jiang Y, et al. Human Embryonic Stem Cell-Derived Cardiovascular Progenitors Repair Infarcted Hearts Through Modulation of Macrophages via Activation of Signal Transducer and Activator of Transcription 6. *Antioxid Redox Signal*. 2019;31:369-86.
10. Zhao P, Zhou W, Zhang Y, Li J, Zhao Y, Pan L, et al. Aminooxyacetic acid attenuates post-infarct cardiac dysfunction by balancing macrophage polarization through modulating macrophage metabolism in mice. *J Cell Mol Med*. 2020;24:2593-609.
11. Wu J, Wang J, Zeng X, Chen Y, Xia J, Wang S, et al. Protein phosphatase 2A regulatory subunit B56beta modulates erythroid differentiation. *Biochem Biophys Res Commun*. 2016;478:1179-84.
12. Wu J, Dong Y, Teng X, Cheng M, Shen Z, Chen W. Follistatin-like 1 attenuates differentiation and survival of erythroid cells through Smad2/3 signaling. *Biochem Biophys Res Commun*. 2015;466:711-6.
